# References of Birth Weights for Gestational Age and Sex from a Large Cohort of Singleton Births in Cameroon

**DOI:** 10.1155/2014/361451

**Published:** 2014-05-12

**Authors:** Jean Dupont Kemfang Ngowa, Irénée Domkam, Anny Ngassam, Georges Nguefack-Tsague, Walter Dobgima Pisoh, Cyrille Noa, Jean Marie Kasia

**Affiliations:** ^1^Department of Obstetrics and Gynecology, Faculty of Medicine and Biomedical Sciences, Yaounde General Hospital, University of Yaounde I, P.O. Box 5408, Yaounde, Cameroon; ^2^Chantal Biya International Reference Centre for Research on HIV/AIDS Prevention and Management, P.O. Box 3077, Yaounde, Cameroon; ^3^Department of Public Health, Faculty of Medicine and Biomedical Sciences, University of Yaounde I, P.O. Box 1364, Yaounde, Cameroon

## Abstract

*Objective.* To establish the percentile charts of birth weights for gestational age and sex within the Cameroonian population. *Methods.* A review of medical records of infants born between January 2007 and December 2011 at the maternities of two hospitals in Cameroon, Central Africa. Multiple pregnancies, births of HIV infected women, stillbirths, and births with major fetal malformations were excluded. The smooth curves of birth weight for gestational age and sex were created using the Gamlss package under R.3.0.1 software. *Results.* The birth weights of 12837 live birth singleton infants born to HIV negative women between 28 and 42 weeks of gestation were analyzed to construct the birth weight curves for gestational age and sex. The smoothed percentile curves of birth weights for gestational age and sex of Cameroonian infants have demonstrated an increasing slope until 40 weeks and then a plateau. There was a varied difference of distribution in birth weights for gestational age between Cameroonian, Botswanan, American, and French infants. *Conclusion.* We established the reference curves of birth weights for gestational age and sex for Cameroonians. The difference in birth weight curves noted between Cameroonian, Botswanan, American, and French infants suggests the importance of establishing the regional birth weight norms.

## 1. Introduction

Birth weight is one of the important indicators used to assess the health of an infant at birth. It is often used as an outcome measure in perinatal research or as an indicator of perinatal health in clinical practice [1]. Gestational age is a good predictor of birth weight and perinatal survival. After the control of the effects of gestational age, birth weight alone has a strong association with perinatal survival [2]. Low birth weight has been defined by the World Health Organization (WHO) as birth weight of less than 2,500 grams. The infants weighing less than 2,500 grams are approximately 20 times more likely to die than those weighing more [3, 4].

Small for gestational age (birth weight < 10th percentile) or large for gestational age (birth weight > 90th percentile) babies may be identified using the centile charts of birth weight for gestational age and sex [2]. For most developing countries, data on birth weight is collected via annual demographic health survey records and birth weight for gestational age is generally unavailable [3, 4]. However, it is well recognized that there is a wide variation in the birth weight for gestational age of infants depending on ethnicity and sex [5, 6]. In a recent study, Matthews et al. [7] reported that preterm Botswana-born infants had higher average birth weights while term Botswana infants had lower birth weights compared to those born in the US. This finding confirms the geographical variability of birth weights.

In Cameroon, the reference curves for birth weight used in clinical practice are those established in the European or American populations [8, 9]. The establishment of specific norms of birth weight for gestational age in a Cameroonian population may be an important step towards identifying infants at higher risk of early morbidity or death. The aim of this study was to establish the percentile charts of birth weights for gestational age and sex within the Cameroonian population.

## 2. Methods

This was a medical record review of infants born between January 2007 and December 2011 at the maternities of the Yaounde General Hospital (YGH) and Yaounde Gyneco-Obstetric and Pediatric Hospital (YGOPH) situated in Cameroon, Central Africa. These hospitals are located in the urban setting of Yaounde and are among the most attended health centers in this town. Yaounde is the political capital of Cameroon; its population is cosmopolitan and representative of all the ethnic groups of this country. Because of their geographical location, the maternities of these two hospitals receive mothers from all ethnic groups of this country. In these maternities, birth records are filled by the midwives.

All live born singleton infants from 28 to 42 weeks of gestational age during the study period were recruited. The gestational age was calculated in completed weeks from the last menstrual period since the use of ultrasound in the dating of pregnancies is not a common practice in our milieu. The cases with major congenital abnormalities, stillbirths, HIV positive mothers, unknown and uncertain gestational age, and incomplete data were excluded from the study. Perinatal data was collected from the delivery registries and filled by midwives. This data included date of delivery, gestational age at birth, singleton birth, birth weight, infant sex, viability, stillbirths, infants with major congenital abnormalities observed, and HIV status of the mother.

The first step of the statistical analysis consisted in cleaning the outliers from the data. We used the method described by Foster and Kecojevié [10] who used a robust regression of birth weight on log transformation of gestational ages to identify the most extreme values. The observations with the big residuals are weighted down, which reflect that they are atypical from the rest of the observations when it comes to fitting such a model. Observations with zero weight are deemed to be extreme and so are then removed from the data as outliers before the construction of the curves. The zero weight does not refer to the body weight but corresponds to the zero influence of the individual in the model used. In the second step, we constructed the gender specific birth weights for gestational age curves. BCPE model with cubic smoothing spline model was used. The BCPE distribution was chosen within a set of distributions (BCPE, LNO, PE, BCT, and JSU) based on the generalised Akaike information criterion (GAIC). The Gamlss function “find.hyper” was used to select the appropriate hyperparameters for mu and sigma and the power parameters.

**Figure 1:**
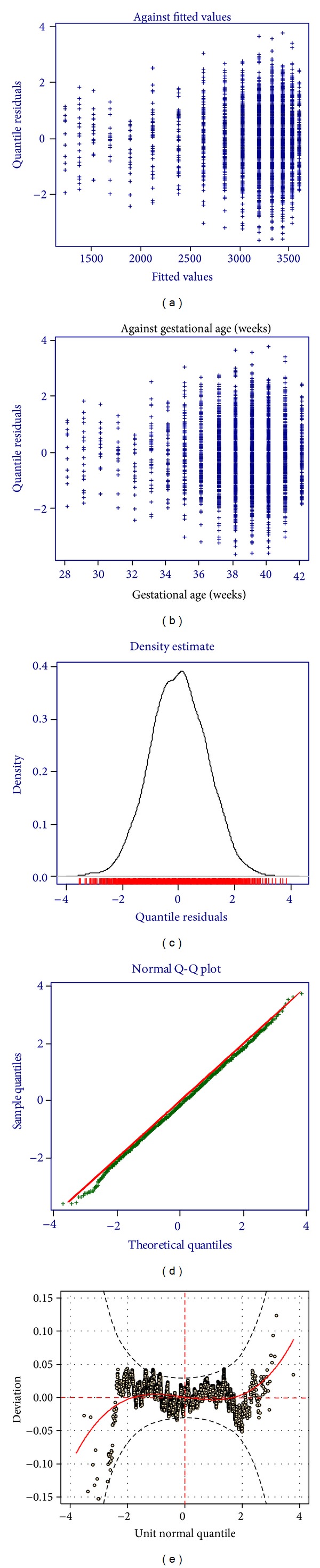
Residual and worm plots of birth weights of male Cameroonian infants.

**Figure 2:**
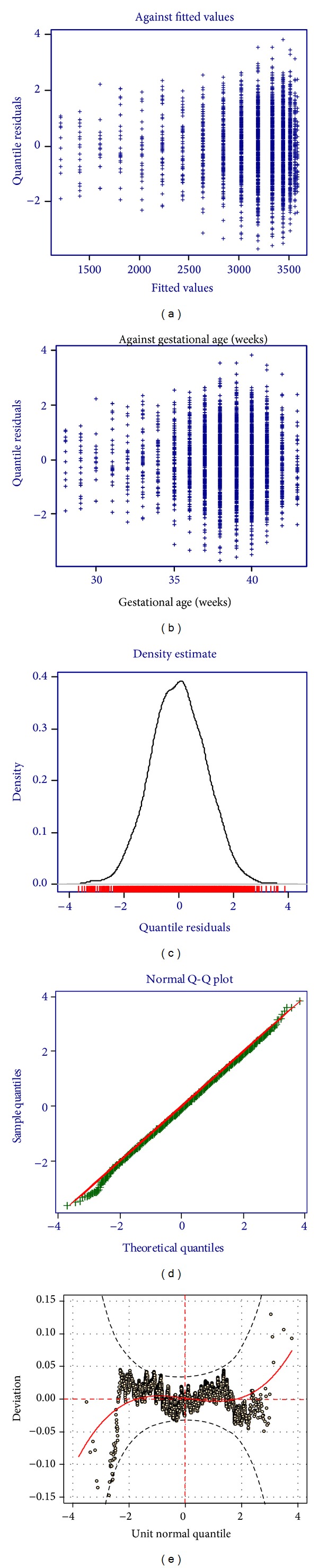
Residual and worm plots of birth weights of female Cameroonian infants.

The smoothed percentile curves for the 10th, 25th, 50th, 75th, and 90th percentiles were created using the Gamlss package under R.3.0.1 software. Worm plots and residual plots were used to assess the normality of the curves for each sex [11] (Figures 1 and 2). The median birth weights of Cameroonian born infants at each gestational age were compared to those of Botswanan and American born infants and birth weights at 10th, 50th, and 90th centiles of Cameroonian infants were compared with those of French infants. The researchers obtained permission to carry out this study from the two hospitals management team represented in each hospital by the head of the medical team.

## 3. Results

**Figure 3:**
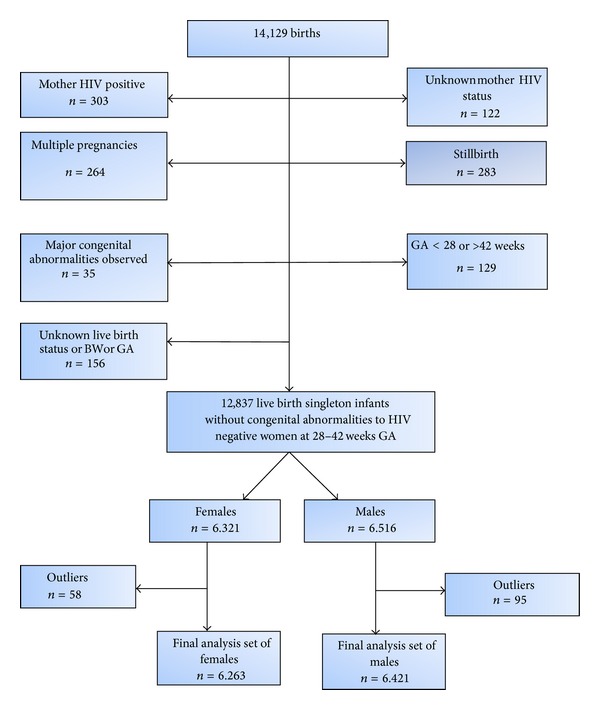
Selection of analysis dataset of births. GA: gestational age; BW: birth weight.

A total of 14129 births were collected as initial dataset, of which 6480 were from the Yaounde General Hospital and 7649 were from the Yaounde Gyneco-Obstetric and Pediatric Hospital. An HIV status was recorded for 99% of the mothers, of which 02.16% (303) were HIV positive. We excluded the births from HIV positive mothers (303); mothers with unknown HIV status (122); births from multiple pregnancies (264); stillbirths (283); births with major congenital abnormalities (35); births at <28 or >42 weeks of gestational age (129); and births with unknown viability status, birth weight, or gestational age (156). After application of these exclusion criteria, we obtained 12837 live birth singleton infants without congenital abnormalities born to HIV negative women at 28–42 weeks of gestational age. Then, 153 outliers were also excluded leaving a final dataset analysis of 6263 for female infants and 6421 for male infants (Figure 3).

Crude birth weights for gestational ages ranging from 28 to 42 weeks for male and female infants are illustrated, respectively, in Tables 1 and 2. The number of infants at each gestational age ranged from 17 at 29 weeks to 1638 at 39 weeks for male infants and from 12 at 29 weeks to 1663 at 39 weeks for female infants. The median gestational age at delivery among all infants was 39 weeks and the median birth weight was 3206 g. The male infants were generally heavier than the female infants for each gestational age with a median birth weight of 3260 g for the males compared to 3160 g for the females (*P* < 0.001). Low birth weight (<2500 g) represented 9.8% of the study population, with 8.3% for male infants and 11.3% for female infants.

**Figure 4:**
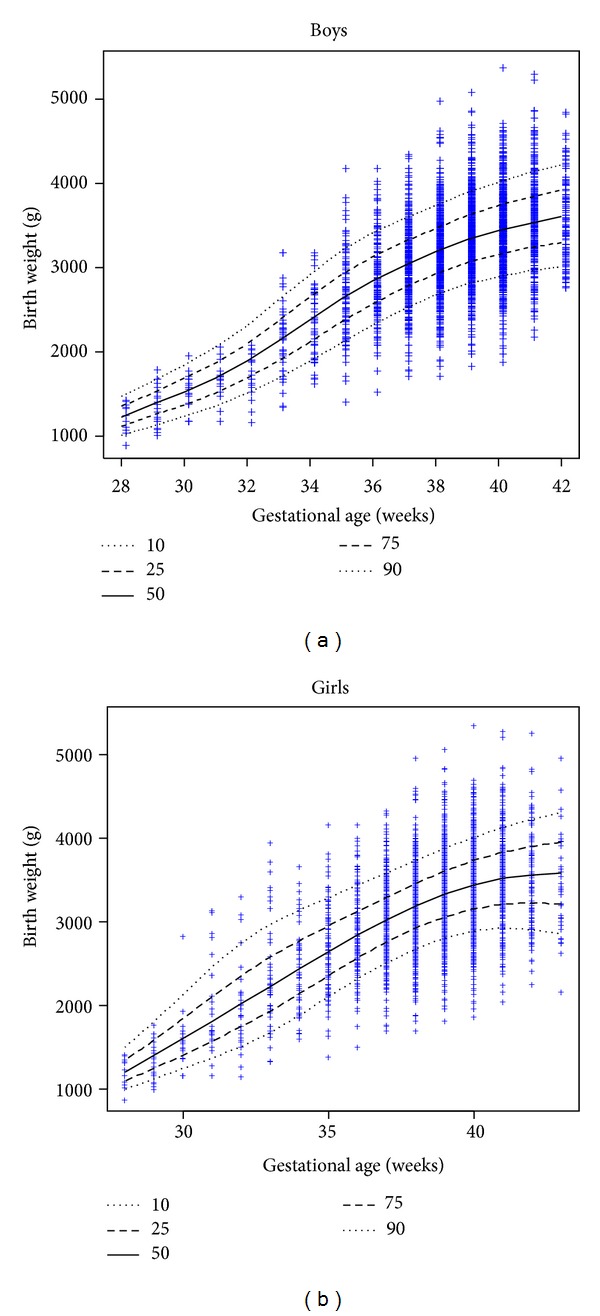
Smoothed percentile curves for the birth weights of male and female Cameroonian infants.

The smoothed percentile curves for the birth weights of male and female Cameroonian infants are represented in Figure 4. These curves reveal an increasing slope until 40 weeks and then a plateau. Tables 3 and 4 display the estimated birth weight percentiles for gestational age for Cameroonian males and females, respectively.

**Figure 5:**
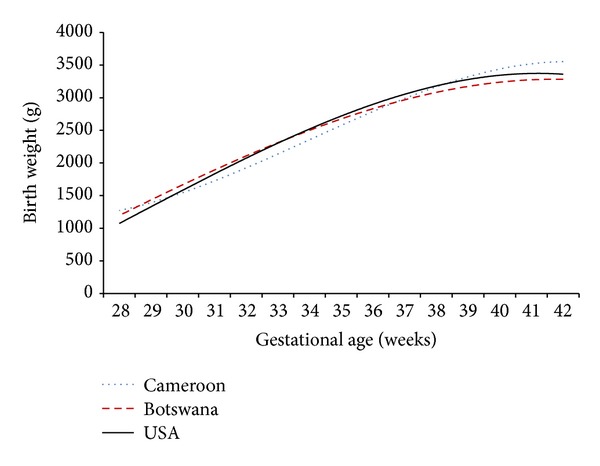
Median birth weights for gestational age of Cameroonian infants compared to those of Botswana and USA infants.

Figure 5 shows the superimposed curves of median birth weight for gestational age constructed from the Cameroon dataset and Botswana and USA data [7]. These curves reveal that Cameroon born infants weighed less than Botswana and USA born infants between 29 and 36 weeks and between 30 and 37 weeks, respectively, and they had a tendency to be heavier thereafter.

**Figure 6:**
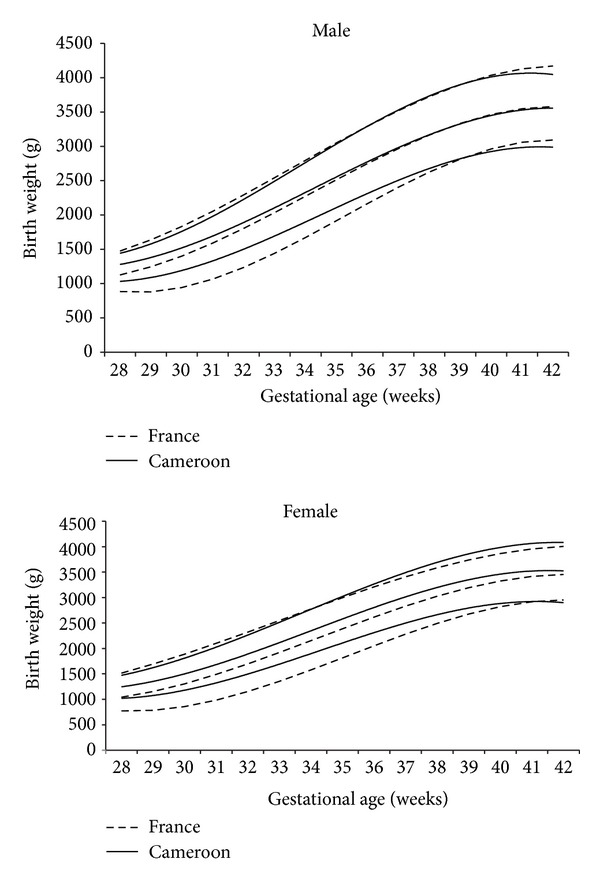
10th, 50th, and 90th percentile curves of birth weights of male and female Cameroonian infants compared to French infants.

Figure 6 shows the 10th, 50th, and 90th percentile curves of Cameroon born infants compared to French born infants for males and females [9]. The curves for male infants reveal that preterm Cameroon born infants seem to be heavier than preterm French babies at 10th and 50th percentiles; however, they tend to be less heavy beyond term. Similarly, the curves for female infants reveal that Cameroon born babies tend to be heavier than the French babies at the 10th and 50th percentiles.

## 4. Discussion

This study describes percentile charts of birth weights for gestational age and sex for live singleton infants born in two hospitals in Yaounde. We believe that this hospital based data is representative of the whole Cameroonian population, since the data was collected in two of the most attended hospitals in Yaounde where the population is a mixture of people from different regions of the country. Equally, the proportion of low birth weight infants of 9.8% in this study is close to the 11% of low birth weight infants estimated in the Cameroonian population in 2003 by the UNICEF based on data of demographic and health survey [3]. This data may serve as a reference for birth weight for gestational age in this population. We believe that these curves of birth weight for gestational age will provide to obstetricians, pediatricians, and researchers a useful tool that is more suitable than American and European references for the Cameroonian population. For clinicians, it improves the classification of individual infants within the following categories: appropriate, small, or large for their gestational age. For researchers, it allows a better classification of groups of infants so as to determine geographical differences, temporal trends, etiologic determinants, and short and long term prognosis.

In most developing countries, the reference of birth weight for gestational age is unavailable. In these countries, there is a lack of national birth weight registers and birth weight is collected via annual demographic health surveys [3]. As a matter of fact, currently used birth weights for gestational age references in most developing countries are those from North America or Western Europe which are not necessarily suitable for the concerned populations [8, 9, 12]. Several studies have revealed the racial and/or ethnic and geographical variation of birth weights, with higher birth weights among European rather than African and Asian infants [5].

The difference in birth weights between Cameroonian born infants and those from Botswana and USA can be explained by the geographical variation due to environmental factors. The small sample size in preterm Cameroonian infants in both sexes and the births from multiple pregnancies included in the Botswana study could skew the data toward the lower birth weights.

We noted a difference in the curves of birth weights for gestational age of Cameroonian infants and French infants. The male Cameroonian infants born at <35 weeks tend to have higher birth weights than French born infants at the 10th and 50th percentiles and there after they seem to be less heavy, while the female Cameroonian infants tend to have at the 10th and 50th percentiles a higher birth weight than French female infants. The difference in birth weights for gestational age between Cameroonian and French infants can be explained by racial and geographical factors and probably the smaller sample size of preterm infants in our study. In this study, the sample size was less than 100 between 28 and 34 weeks which did not assure the best statistical strength.

One immediate impact of dating pregnancy using the last menstrual period is the misclassification of gestational age with risk of an increase or a decrease in birth weights for gestational age. This inaccuracy of dating pregnancy using the last menstrual period is due to recall bias, varied menstrual cycles, irregular cycles, and misinterpretation of bleeding at the time of embryo implantation [13]. The gestational age in our study was estimated from the last menstrual period unlike in the French study where it was calculated from ultrasound dating. One immediate impact of using specific birth weight charts for different ethnic or racial groups is that it permits the identification of the true proportion of macrosomic babies (birth weight above 90th percentile) and small for date babies (birth weight under 10th percentile) born from mothers with complications such as gestational diabetes, preeclampsia, malaria, and anemia.

However, this study has some limits. Our data was collected from two hospitals in the urban area; thus the rural population was not well represented. Gestational age may be inaccurate when estimated from the last menstrual period. Parity was not taken into consideration when constructing our reference curves. To minimize these errors, we attempted to correct the data by identifying and excluding the outliers.

## 5. Conclusion

Our study established the birth weight percentile charts for gestational age and sex based on the Cameroonian singleton births. These charts represent the Cameroonian population better than the European or American charts. The differences in curves of birth weights for gestational age observed between the Cameroonian, Botswanan, American, and French populations confirm the geographical variation of birth weights and suggest the importance of establishing the regional birth weight norms. However, there is need to carry out a large multicentric study which will be more representative of the urban and rural populations in our country.

## Figures and Tables

**Table 1:** Distribution of crude birth weights for gestational age for male Cameroonian infants.

Gestational age (weeks)	*n*	Birth weights (grams)				
		Mean	SD	Median	Minimum	Maximum
28	29	1109	181	1090	750	1450
29	17	1383	237	1400	1030	1810
30	22	1557	330	1555	1050	2620
31	28	2016	593	1740	1200	3180
32	30	1963	511	1965	1050	3130
33	59	2381	642	2250	1360	4050
34	67	2398	491	2300	1640	3700
35	126	2689	477	2650	1420	4200
36	245	2882	443	2860	1550	4200
37	607	3048	421	3040	1730	4370
38	1234	3215	412	3200	1740	5000
39	1638	3347	429	3330	1860	4880
40	1563	3447	445	3450	1900	4980
41	635	3541	457	3540	2080	4900
42	161	3535	519	3600	2300	4875

SD: standard deviation.

**Table 2:** Distribution of crude birth weights for gestational age for female Cameroonian infants.

Gestational age (weeks)	*n*	Birth weights (grams)				
		Mean	SD	Median	Minimum	Maximum
28	23	1053	207	1030	700	1500
29	12	1342	339	1275	900	1900
30	18	1581	435	1470	1000	2650
31	25	1940	587	1700	1200	3050
32	35	1957	461	1850	1130	3200
33	53	2211	537	2100	1300	3450
34	72	2262	476	2273	1260	3400
35	100	2537	521	2465	1500	3880
36	224	2759	489	2715	1640	4330
37	538	2937	449	2900	1700	4725
38	1109	3115	431	3100	1700	4860
39	1663	3202	401	3200	2000	4680
40	1531	3318	427	3300	1900	4900
41	693	3396	449	3400	1890	4630
42	167	3374	472	3350	2110	4830

SD: standard deviation.

**Table 3:** Estimated birth weight percentiles for gestational age for male Cameroonian infants.

Gestational age (weeks)	Estimated male percentile birth weights (grams)								
	C3	C5	C10	C25	C50	C75	C90	C95	C97
28	906	946	1008	1112	1227	1348	1470	1549	1602
29	1002	1048	1120	1240	1372	1512	1652	1743	1804
30	1099	1152	1234	1371	1522	1682	1841	1945	2015
31	1202	1263	1357	1514	1688	1871	2054	2172	2252
32	1323	1393	1502	1685	1887	2098	2310	2447	2540
33	1474	1555	1681	1892	2125	2369	2613	2770	2876
34	1653	1743	1884	2120	2379	2651	2921	3094	3212
35	1855	1952	2103	2354	2630	2917	3202	3384	3507
36	2063	2162	2316	2570	2848	3136	3420	3601	3723
37	2258	2356	2507	2757	3029	3310	3585	3760	3878
38	2425	2523	2674	2922	3192	3469	3740	3912	4027
39	2548	2649	2803	3057	3332	3614	3889	4063	4180
40	2627	2732	2892	3156	3441	3734	4018	4198	4318
41	2686	2794	2961	3235	3531	3834	4128	4313	4437
42	2730	2843	3015	3298	3603	3916	4218	4409	4536

**Table 4:** Estimated birth weight percentiles for gestational age for female Cameroonian infants.

Gestational age (weeks)	Estimated female percentile birth weights (grams)								
	C3	C5	C10	C25	C50	C75	C90	C95	C97
28	902	936	991	1087	1201	1336	1500	1625	1719
29	1004	1048	1119	1246	1397	1579	1801	1970	2099
30	1098	1153	1243	1405	1601	1839	2129	2350	2517
31	1191	1258	1369	1569	1812	2107	2462	2730	2932
32	1301	1380	1509	1741	2021	2355	2749	3039	3253
33	1448	1536	1678	1930	2229	2576	2971	3253	3457
34	1639	1732	1880	2138	2436	2771	3138	3392	3572
35	1858	1952	2101	2356	2643	2957	3289	3513	3669
36	2076	2171	2318	2566	2841	3136	3438	3638	3775
37	2272	2367	2513	2757	3023	3303	3585	3768	3893
38	2435	2531	2679	2924	3190	3465	3738	3913	4031
39	2552	2653	2807	3060	3333	3613	3888	4062	4178
40	2619	2726	2890	3157	3444	3735	4017	4194	4313
41	2632	2749	2926	3215	3522	3833	4129	4313	4436
42	2576	2706	2904	3225	3564	3904	4224	4421	4551
